# Germinal Center Cells Turning to the Dark Side: Neoplasms of B Cells, Follicular Helper T Cells, and Follicular Dendritic Cells

**DOI:** 10.3389/fonc.2020.587809

**Published:** 2021-01-15

**Authors:** Rosario Munguía-Fuentes, Raúl Antonio Maqueda-Alfaro, Rommel Chacón-Salinas, Leopoldo Flores-Romo, Juan Carlos Yam-Puc

**Affiliations:** ^1^ Departmento de Ciencias Básicas, Unidad Profesional Interdisciplinaria en Ingeniería y Tecnologías Avanzadas, Instituto Politécnico Nacional, UPIITA-IPN, Mexico City, Mexico; ^2^ Department of Cell Biology, Center for Advanced Research, National Polytechnic Institute, Cinvestav-IPN, Mexico City, Mexico; ^3^ Departamento de Inmunología, Escuela Nacional de Ciencias Biológicas, Instituto Politécnico Nacional, ENCB-IPN, Mexico City, Mexico; ^4^ Institute of Immunology and Immunotherapy, College of Medical and Dental Sciences, University of Birmingham, Birmingham, United Kingdom

**Keywords:** peripheral T-cell lymphomas, angioimmunoblastic T cell lymphoma, follicular T-cell lymphoma, follicular dendritic cell sarcomas, follicular lymphoma, Burkitt lymphoma, diffuse large B cell lymphoma

## Abstract

Gaining knowledge of the neoplastic side of the three main cells—B cells, Follicular Helper T (Tfh) cells, and follicular dendritic cells (FDCs) —involved in the germinal center (GC) reaction can shed light toward further understanding the microuniverse that is the GC, opening the possibility of better treatments. This paper gives a review of the more complex underlying mechanisms involved in the malignant transformations that take place in the GC. Whilst our understanding of the biology of the GC-related B cell lymphomas has increased—this is not reviewed in detail here—the dark side involving neoplasms of Tfh cells and FDCs are poorly studied, in great part, due to their low incidence. The aggressive behavior of Tfh lymphomas and the metastatic potential of FDCs sarcomas make them clinically relevant, merit further attention and are the main focus of this review. Tfh cells and FDCs malignancies can often be misdiagnosed. The better understanding of these entities linked to their molecular and genetic characterization will lead to prediction of high-risk patients, better diagnosis, prognosis, and treatments based on molecular profiles.

## Introduction

The germinal center (GC), a specialized microstructure with a high rate of cell division, is the site where antigen-driven somatic hypermutation (SHM) occurs (1, 2), a process that ultimately will produce high-affinity antibodies during adaptive immune responses (3). Over weeks, memory B cells and high-affinity antibody producing plasma cells will generate from GCs, which are necessary to protect against invading microorganisms (4). However, the more potent the immune response, the greater the risk of autoreactivity or malignancy. This is particularly relevant for the GC, where B cells may have an unfavorable outcome driving to lymphomagenesis. Importantly, most of B−cell lymphomas originate from GC B cells (5–7).

**Figure 1:**
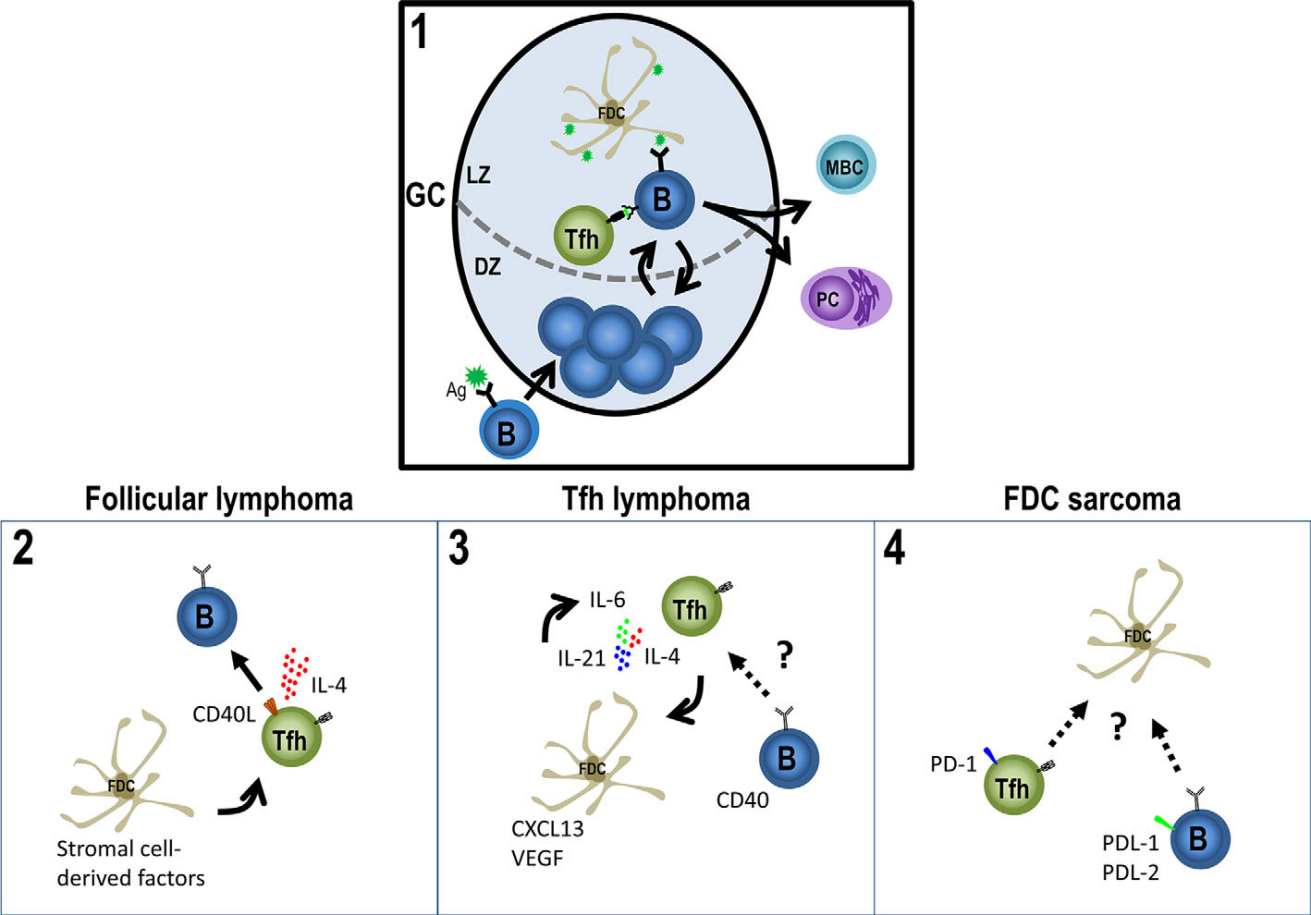
Germinal Center-derived malignancies’ players. The GC (1) is the site for B cell affinity maturation for the antigen through somatic hypermutation (in the DZ) and for antigen-driven selection of B cells which have improved their affinity (in the LZ). To success the reaction, the help from Tfh cells is fundamental, as well as, the presence of the antigen on iccosomes on FDCs. Eventually, GC B cells will differentiate to PCs or MBCs. However, the GC response may have a detrimental role, the development of malignancies from their three main players: B-cells, Tfh cells and FDCs. Follicular lymphoma cells (2) derive from follicles partly resembling normal GCs and depend on Tfh cells and FDCs to survive, while Tfh cells provide a high production of IL-4 and CD40L, FDCs provide a scaffold attracting FL cells and Tfh cells around them. (3) Infiltration of different immune cells and the proliferation of FDCs and HEV in AITL are probably caused by a stimulatory niche, secreting IL-21, IL-4 or/and IL-6, CXLC13 and VEGF, promoting a loop of Tfh cell generation and FDCs growth. (4) Tfh and Treg cells seem to be enriched in FDC sarcomas with high levels of PD-1 and its ligands PD-L1 and PD-L2 and the B/T cells mixed with the neoplastic population, altogether supporting the neoplastic niche and the evasion of effector immune cells. See text for further details.

To succeed during GC reactions, B cells need the help of other crucial cells, such as Follicular Helper T (Tfh) cells and follicular dendritic cells (FDCs). Here, we focus on one dark side of GCs: malignancies derived from their aforementioned three players, B-cells, Tfh cells, and FDCs (**Figure 1**), with greater emphasis on Tfh lymphomas and FDC sarcomas (**Table 1**).

**Table 1:** Lymphomas of Follicular Helper T (Tfh) cells and sarcomas of follicular dendritic cells (FDCs).

		Incidence and OS	Clinical features	Morphology	Immunophenotype	Genetic profile	Treatment
**Malignancies of Tfh cells origin**							
AITL	15-30% of non-cutaneous T-cell lymphomas and 1-2% of all non-Hodgkin lymphomas. Poor prognosis overall. Median survival < 3 years.		Systemic symptoms, lymphadenopathy, hepatosplenomegaly, polyclonal hypergammaglobulinemia. Frequent skin rash. Hemolytic anemia, pleural effusion, arthritis, and ascites are also common.	Polymorphous infiltrate with small- to medium-size neoplastic cells. **-Pattern 1.** Neoplastic cells surrounding follicles. **Pattern 2.** Neoplastic cells in the expanded paracortex. **Pattern 3.** Diffuse with nearly total LN architecture altered. Expanded FDCs meshworks. Prominent vascularity.	Pan-T cell markers (CD2, CD3, and CD5) with Tfh cell markers: CD4, CD10, CXCL13, ICOS, BCL6, PD1 (60-100% of cases).	Trisomies of chromosomes 3, 5, and 21; gain of X; and loss of 6q (90% cases). Mutations of *IDH2* (20- 30%), *TET2* (50―80%), and *DNMT3A* (20―30%), *RHOA* (60―70%).	Multiagent chemotherapy regimen, CHOP. Steroids. Stem cell transplantation.
FTCL	Unknow incidence, accounts for < 1% of all T cell neoplasms. Aggressive course. OS not well characterized. 50% patients dye within 24 months of diagnosis.		See AITL clinical symptoms. Reports of few patients with localized disease and/or no B cell- related symptoms.	Monotonous lymphoid cells with abundant pale cytoplasm and round nuclei. Nodular/follicular proliferation. **Pattern 1.** GC-like growth. **Pattern 2.** nodular FL-like growth. No expanded FDCs meshworks nor HEV proliferation.	Tfh cell markers. See above.	t(5;9) (q33;q22) (ITK/SYK) (20% cases). *TET2, RHOA and DNMT3A* mutations (75, 60 and 25% of cases, respectively. Not largely studied).	See AITL.
Nodal PTCL with Tfh cell phenotype	————–		Overlaps with AITL.	Diffuse infiltration without vascular proliferation or expansion of FDCs meshwork.	CD4+ T cells with two (preferred three) Tfh cell markers.	Mutations shared with AITL	See AITL.
**Malignancies of FDCs**							
FDC sarcoma	Unknow. Rare disorder. Constitutes <0.4% of soft tissue sarcomas. 2-year survival rates: Early disease: 82% Local advanced disease: 80% Distant metastatic: 42%		Systemic symptoms are uncommon. Often large tumors (mean size of 7cm). Painless, slow-growing mass lesion.	Spindled to ovoid cells forming different patterns (storiform arrays, fascicles, whorls, diffuse sheets, or vague nodules).	One or more FDCs markers: CD21, CD23, CD35. CXCL13 and podoplanin (not specific). Clusterin (strongly positive). FDCSP, serglycin (SRGN) and PD-L1.	Limited studies. BRAF V600E mutation (0-19% of cases). Alterations in tumor suppressor genes.	Complete surgical excision. No clear benefit of radiotherapy and chemotherapy.

AITL, angioimmunoblastic T cell lymphoma; LN, Lymph node; FDCs, Follicular Dendritic cells; Tfh, T follicular helper; FTCL, Follicular T cell lymphoma; GC, germinal center; FL, Follicular lymphoma; PTCL, peripheral T cell lymphoma. CHOP; cyclophosphamide, doxorubicin (or hydroxydaunorubicin), vincristine (also known as Oncovin^®^) and prednisolone.

## The Germinal Center

GCs arise from proliferating B cells in the follicles of peripheral lymphoid tissues during T cell-dependent antibody responses. Naïve B cells encountering their antigen migrate to the T-B border, where they become fully activated during interaction with cognate CD4+ T cells (3, 4, 8–12). The engagement of CD40 by CD40L (CD154) represents the major component of the T cell help. Activated B cells can then either differentiate rapidly into antibody-secreting plasma cells in specialized extra-follicular niches or mature their affinity for the antigen into GC reactions, a microstructure of B cells, in a high-rate of cell division, Tfh cells and a network of FDCs (3, 13, 14). There, B cells begin to proliferate rapidly giving rise to the distinctive structure of the GC: a dark zone (DZ) of centroblast proliferating B cells and a light zone (LZ) with higher frequencies of smaller, non-dividing centrocytes. GC DZ B cells undergo SHM, and those cells that improved the affinity for the antigen are selected in the LZ to eventually differentiate into memory B cells or plasma cells (3, 4, 15, 16).

## The Dark Side of GC B Cells

The GC response, beneficial for the host during immune responses against invading pathogens, may have a detrimental role, the development of malignancies. B cells inside GC reactions are mutating at much higher rates than in any other site in the body (17), these mutations might turn B cells into a dark side, B cell lymphomas. Except the relatively rare lymphoblastic and mantle-cell lymphoma subtypes, B cell non-Hodgkin lymphomas (B−NHLs)—including diffuse large B cell lymphoma (DLBCL), follicular lymphoma (FL) and Burkitt lymphoma (BL)—are derived from GC B cells. This can be demonstrated by the presence of SHM in the immunoglobulin genes, together with histological, immunophenotypic, and gene expression characteristics (5, 18–23).

Our understanding of the molecular mechanisms driving GC lymphomas has increased due to next-generation sequencing (24). Gene translocations targeting MYC, BCL2 and BCL6, as well as the disruption of the epigenome, predominantly driven by somatic mutations within KMT2D, CREBBP, EZH2, and linker histones, have been well-established (25–29). The molecular and genetic characterization of these diseases will lead to prediction of high-risk patients and treatments based on molecular profiles (24).

## Diffuse Large B Cell Lymphoma

DLBCL and FL are the two most common forms of GC NHLs. With a greater degree of genetic heterogeneity than FL, DLBCL can be divided into at least two major subtypes: GC B cell (GCB)-like and activated B cell (ABC)-like DLBCL (24, 25, 30). Whole exome sequencing has allowed the study of recurrent mutations and the characterization of new genetic DLBCL subtypes. A recent study identified five genomic clusters based on the enriched genetic feature of each group (31). MYD88 cluster [with MYD88 (L265P), PIM1, CD79B and ETV6 mutations] were strongly associated with ABC subtype. Three clusters were associated to GCB subtype (BCL2, SOCS1/SGK1, and TET2/SGK1). The BCL2 cluster showed mutations of EZH2, BCL2, CREBBP, TNFRSF14, KMT2D, and MEF2B. The SOCS1/SGK1 cluster with mutations in SOCS1, CD83, SGK1, NFKBIA, HIST1H1E, and STAT3; and the TET2/SGK1 cluster characterized by mutations including TET2, SGK1, KLHL6, ZFP36L1, BRAF, MAP2K1, and KRAS. A NOTCH2 cluster with mutations on NOTCH2, BCL10, TNFAIP3, CCND3, SPEN, TMEM30A FAS, and CD70 showed a mixture of ABC, GCB and unclassified DLBCL. This study correlated with two recent studies classifying the disease (32, 33). Importantly, patient outcome was evaluated with the worst prognosis in the MYD88 group (42% 5-year overall survival, OS). Patients within the GCB-associated clusters had better 5-year OS (> 60%) while NOTCH2 cluster had intermediate survival (53.6% 5-year OS). Patient outcome correlated with previous studies (24, 34–38).

## Follicular Lymphoma

FL is the most frequent indolent and incurable NHL. Over time FL may progress to DLBCL, with a more aggressive clinical course requiring more aggressive treatment (39, 40). Malignant cells morphologically resemble the two B cell subsets found in reactive GCs (centrocytes and centroblasts). Low-grade FL cases (grade 1–2) contain <15 centroblasts per high-power microscopic field (40 x objective, 0.159 mm^2^) while grade 3 contains >15 centroblasts, evaluated in 10 different follicles. Grade 3 FL is further separated in 3A with a background of centrocytes present or grade 3B with follicles composed entirely of centroblasts. A diffuse pattern of 3B grade cells is compatible with DLBCL diagnosis (41, 42). The molecular pathogenesis of FL includes the high recurrence of two mutations: chromosomal translocations that lead to the ectopic expression of BCL2 and somatic mutations in the histone methyltransferase MLL2 (also known as KMT2D) (43). Also, histone modifiers such as EZH2, CREBBP and EP300 are frequently altered in FL (44–46). While BCL2 translocation is thought to occur in B-cell precursors in the bone marrow, the translocation is found also in healthy humans (40%) (47). This supports the hypothesis that the translocation is necessary, but not sufficient for FL and probably lymphomagenesis is consequence of antigen stimulation (48, 49).

FL derives from follicles partly resembling normal GCs, with the FL cells depending on Tfh cells and FDCs. While Tfh cells provide a high production of IL-4 and CD40L as survival factors of FL cells, FDCs provide a scaffold attracting FL cells and Tfh cells around them. FDCs also contributes with a positive feedback through the overexpression of stromal cell-derived factors, supporting the abnormal production of IL-4 (50, 51).

## Burkitt Lymphoma

BL is a highly aggressive NHL associated with Epstein-Barr virus (EBV), human immunodeficiency virus (HIV) or Plasmodium infection. Three clinical variants of BL are recognized: endemic BL, sporadic BL and immunodeficiency-associated BL. Whilst EBV and Plasmodium are associated to endemic and sporadic BL, HIV is associated to the immunodeficiency-related variant (52, 53). BL derives from DZ GC B cells, as indicated by its genetic profile (20, 23, 54). Although the potential pathological role of EBV in BL it is still controversial, the virus is present in all BL cases. Aberrant expression of MYC and the BCR-induced PI3K signalling pathway activation are genetic alterations that are common in BL (23, 55, 56).

## Follicular Helper T Cells

Fundamental studies by Mitchison in the 1970’s established the essential role of T helper cells in antibody responses. Hapten-protein carrier conjugates revealed that carrier-specific T cells were necessary for the maturation of hapten-specific B cells [(57) and reviewed in Ref (10)]. Then, it was described that this help from T cells consisted in co-stimulatory signals through CD40 ligand (CD40L) to B cells leading them to proliferate, differentiate, and antibody class-switching (10, 58). T cells in the T-B border that have undergone T-B interactions can migrate inside the follicles as Tfh cells, afterwards making cognate interactions with GC B cells within the GC reaction. T cell help into GCs are needed to maintain the reaction (59–63).

At present, a combination of markers is needed to identify Tfh cells as a distinct population. Tfh cells differentiate from the classical CD4+ T cell subpopulation and share plastic characteristics with other CD4+ helper T cells until they engaged in GC reactions. Inside GCs, the expression of typically described Tfh cells associated-molecules—CXCR5, PD-1, BCL6, BTLA4, ICOS (inducible T cell costimulator) and SAP—is upregulated whilst CD127, PSGL1, and EBI2 are downregulated (64–66). When these molecules are low-intermediate expressed, particularly CXCR5, PD-1, ICOS and SAP, define stage known as pre-GC Tfh cells (10, 66–69).

## Follicular Helper T Cells in Malignancy

Peripheral T-cell lymphomas (PTCLs) are generally described as diverse and aggressive malignancies with unfavorable therapeutic outcomes (70). Nodal T-cell lymphomas with Tfh-cell phenotype are classified into three diseases: angioimmunoblastic T cell lymphoma (AITL), follicular T-cell lymphoma (FTCL) and nodal PTCL with Tfh cell phenotype. AITL is an aggressive rare tumor with a 5-year survival of only 33%, first described as a distinct clinic-pathologic entity in the 1970s and is the best well-established subtype of mature PTCL (71, 72). The tumors contain neoplastic Tfh cells expressing BCL-6, CD10, CXCL13, PD-1, ICOS, SAP, and CXCR5 (10, 73–75). TET2, DNMT3A and IDH2 mutations have been detected in about 80% (76–78), 20–30% (76, 79, 80), and 20–30% of the cases (81), respectively. The mechanism of action described for these molecules is by dysregulating DNA methylation (70). A missense mutation in RHOA GTPase is detected in 50–70% AITL patients (77, 78, 82, 83). It has been described that some primary cutaneous T cell lymphomas also originate from neoplastic cells that express Tfh cells markers, and can also induce the typical rosettes found in AITL (84).

Representative clinical symptoms of AITL are generalized lymphadenopathy, hepatosplenomegaly, fever, effusion/ascites and skin rash. The incidence is low without sex predilection, affecting advanced-age individuals (median age of diagnosis 65 years) (72). Characteristically, lymph nodes acquire an effaced architecture, with only a few benign follicles been retained. A typical feature is to find infiltration beyond the capsule of the lymph node, with a preserved but enlarged subcapsular sinus; also high endothelial venules (HEV) and FDCs proliferate (71). Infiltration of other cells include: B cells, plasma cells, eosinophils, histiocytes and epithelioid cells. Active EBV infection can be found in most large B cells, whilst the malignant Tfh cells do not (72).

Cytological diagnosis of AITL is usually difficult and both reactive and lymphomatous processes need to be discarded. Combination of conventional cytology, immunocytochemistry and flow cytometry is needed to make an accurate diagnosis (85).

AITL, a lymphoma with poor prognosis, is often refractory to chemotherapy or relapses. Due to the unfavorable outcomes for PTCL patients treated with chemotherapy alone, autologous stem cell transplantation (SCT), as a consolidation treatment for first-line therapy or salvage therapy for relapse/refractory PTCL patients, may be an option. On the other hand, some relapse/refractory AITL patients may benefit from allogenic-SCT, presumably because of graft-versus lymphoma effects (74).

FTCL presents clinical and immunophenotype features of AITL but differs histologically. Two patterns have been described, one shows a GC-like growth with IgD+ B cells surrounding the neoplastic cells and the second resembles a FL-like pattern with malignant cells forming nodules. Another difference with AITL is the absence of proliferation of HEVs and FDCs (86).

While TET2, RHOA and DNMT3A mutations have been shown in both, AITL and FTCL, there is no evidence of IDH2 mutation in FTCL (87). Also, 20% of cases show a t(5;9) (q33;q22) (ITK/SYK) translocation but studies are limited (88).

The study and characterization of normal Tfh cells phenotyping led to the recognition and classification of previously diagnosed PTCLs-NOS (Not otherwise specified) to Nodal PTCLs with Tfh cell phenotype (84). While clinical, phenotypic, pathological, and genetic features overlap with AITL, further research is needed to include this neoplasm within the spectrum of one entity. Differences with AITL include the absence of expansion of HEVs and FDCs while histological differences from FTCL are due to the diffuse pattern of Nodal PTCLs with Tfh cell phenotype (86, 89).

## Tfh Lymphomas and the Interaction With Their Niches

The interaction of neoplastic Tfh cells and their niches has not been extensively studied. The reported infiltration of different immune cells and the proliferation of FDCs and HEV in AITL are probably caused by a stimulatory niche but the underlying mechanisms are still unknown. Some signals present in a normal counterpart niche like IL-21, IL-4, or/and IL-6 are over-expressed in AITL creating a loop of Tfh cell generation and FDCs growth (84, 90, 91). Also, this microenvironment could explain in part the depletion of Treg cells in AITL, an important population for suppressing Tfh cells in immune responses (92). Regulatory CAR T cells therapy might be a potential treatment to re-establish a favorable microenvironment.

Although neoplastic cells in FTCL show a GC- or follicle-growth pattern, the low incidence of this malignancy has made difficult the in-depth study of their interaction with resident cells. Understanding the crosstalk between neoplastic cells and their niche would definitely potentiate the development of more rationale treatments.

## Follicular Dendritic Cells: Origin and Function

Originally discovered by Alexander Maximow and subsequently termed FDCs by Steinman et al. in 1978, FDCs are critical participants in the GC reaction (93–96).

FDCs are stromal cells residing exclusively in B cell follicles, where they play a key role supporting B cell homeostasis and maintaining the follicular architecture. They are essential promoting robust humoral immune responses through the retention of antigens within immune complexes (ICs) over long periods. For this, FDCs express complement receptors (CRs)-1 and -2 and can be induced to express Fc-gamma receptor (FcyR) IIb (93, 97). Lymphoid organs lacking B cells or tumor necrosis factor (TNF) or lymphotoxin (LT) are devoid of FDCs (98–100). Mice lacking stromal CR1 and CR2 have reduced T-dependent antibody responses (93, 98, 101). ICs are released in FDCs-derived iccosomes, then cognate GC B cells can acquire antigen and present it to Tfh cells. FDCs also support the proliferation of GC B cells enhancing antibody production (93, 98).

FDCs are a subset very different from conventional DCs (cDCs). FDCs originate from stromal cells: it has been shown that in the spleen, FDCs come from vascular mural cells but in the lymph nodes, FDCs come from marginal reticular cells (MRCs) (102, 103). Nowadays, it is suggested that different stromal cells of secondary lymphoid organs—including FDCs and MRCs—are generated from one and the same precursor (93).

Functionally, whereas cDCs activate naïve T cells by presentation of processed antigens *via* major histocompatibility complex (MHC) molecules, FDCs show unprocessed antigens, trapped in ICs, to GC B cells. In addition, FDCs secrete the signalling molecule Mfge8 which has been shown to be essential in controlling the removal of apoptotic GC B cells. It has been suggested that FDC-mediated phagocytosis of apoptotic GC B cells might play an important role in avoiding autoimmunity (93).

## Follicular Dendritic Cells Turning to the Dark Side: FDC Sarcoma

Termed FDC sarcomas, the first reported cases of tumors derived from FDCs occurred in cervical lymph nodes. FDC sarcoma is classified as a distinct entity by the World Health Organization (WHO) under histiocytic and DC neoplasms Classification of Tumours. It is described as a neoplastic proliferation of spindled to ovoid cells with morphologic and immunophenotypic characteristics similar to those of normal FDCs. Despite the fact that their histopathological, morphological and clinical features have been described relatively in detail, their clinical course is unpredictable and no specific treatment is available (95, 104).

While FDC sarcomas do not have gender predilection, it mainly occurs during adulthood (median age in the fifth decade). Interestingly, a very rare and distinct variant of FDC sarcoma consistently associated with the EBV, termed inflammatory pseudotumor-like variant of FDC sarcoma, is more prevalent in females. Approximately 10–20% of FDC sarcoma cases have presented or concur with Castleman disease, a rare and non-malignant lymphoproliferative disorder, typically the hyaline vascular variant (104).

We now know also that FDC sarcomas can involve any anatomical area besides nodal sites. FDC sarcomas generally appear as a slow growing mass, an asymptomatic and painless cervical lymphadenopathy (95, 104, 105). Nearly a third of FDC sarcoma cases arise in extranodal sites: tonsils, skin, mediastinum, gastrointestinal tract and soft tissue (104, 106, 107). Furthermore, it seems there is an association between FDC sarcoma and the autoimmune diseases, paraneoplastic pemphigus and myasthenia gravis (104, 108–111).

Histopathology and cytomorphology of FDC sarcomas are characteristic, however their identification may be difficult and additional confirmation with immunohistochemical studies is frequently necessary. FDC sarcomas generally present the immunophenotype of normal FDCs, being positive for: CD21 (CR2), CD23 (Fc epsilon RII) and CD35 (CR1) (104, 112, 113). Clusterin and podoplanin are other molecules shown to have high sensitivity for FDC sarcomas (114–116). Clusterin shows strong positivity with weak to no expression in other DC tumors (114).

Especially when arising from extranodal sites, FDC sarcoma can often be misdiagnosed (106, 117). Then, differential diagnosis is needed, including interdigitating DC sarcoma, thymoma, spindle cell carcinoma, metastatic undifferentiated carcinomas, malignant melanoma and gastrointestinal stromal tumor (GIST) (104).

Clinical courses of FDC sarcomas are not consistent and consequently, treatment schemes are variable. Complete surgical resection seems to be the treatment of choice for both primary and recurrent lesions, with unclear benefits from radiation and chemical therapies (111, 118).

## FDC Sarcoma and Its Interaction With Lymphocytes

Being a very uncommon neoplasm, the in-depth study of FDC sarcomas have been difficult and almost neglected. Although it has been described an enrichment of Tfh and Treg cells in FDC sarcomas compared to other mesenchymal tumors (119), the interaction of malignant FDCs with other lymphocytes and other resident cells has not yet been studied. High levels of PD-1 and its ligands PD-L1 and PD-L2 (119) and the B/T cells mixed with the neoplastic population (120, 121) could point to a feedback from these lymphocytes to support the neoplastic niche and the evasion of effector immune cells.

## Concluding Remarks

As discussed in this review, although lymphomas from GC B cells are explored in more detail and better understood, Tfh lymphomas and FDCs sarcomas need more attention.

Tfh lymphomas diagnosis is challenging, requiring multimodality methods including conventional cytology, immunohistochemistry, and flow cytometry. Usually with a poor prognosis, treatments need to be combined, frequently with unfavorable outcomes.

On the other hand, FDC sarcomas can often be misdiagnosed and differential diagnoses are needed. With variable clinical courses and unspecific and heterogeneous treatment at present, surgical resection is the treatment of choice.

Greater knowledge of the normal GC microuniverse will undoubtedly provide insights on its neoplastic side, allowing us better diagnosis, treatment, prognosis, and monitoring, the better to improve the quality of life of patients.

## In Memoriam

Dedicated to the memory of LF-R, a brilliant Mexican immunologist who inspired many generations of scientists through his passion. His legacy will last forever.

## Author Contributions

Conceptualization, LF-R and JY-P. Writing—Original Draft Preparation, RM-F, RM-A, and JY-P. Writing—Review and Editing, RM-F, RM-A, RC-S, LF-R, and JY-P. Supervision, LF-R and JY-P. All authors contributed to the article and approved the submitted version.

## Funding

This work was supported by a grant from the National Council for Science and Technology-CONACYT Mexico (221102) to LF-R.

## Conflict of Interest

The authors declare that the research was conducted in the absence of any commercial or financial relationships that could be construed as a potential conflict of interest.
